# TYRO3 promotes tumorigenesis and drug resistance in colorectal cancer by enhancing the epithelial-mesenchymal transition process

**DOI:** 10.18632/aging.204656

**Published:** 2023-04-14

**Authors:** Xinyu Shao, Yibin Sun, Kaiqiang Zhong, Jinrong Gu, Yang Yu, Tong Hu, Xiaoyi Kuai, Yechen Xing

**Affiliations:** 1 Department of Gastroenterology, The Affiliated Suzhou Hospital of Nanjing Medical University, Suzhou Municipal Hospital, Gusu School, Nanjing Medical University, Suzhou, Jiangsu 215000, China; 2 Department of General Surgery, The First Affiliated Hospital of Soochow University, Suzhou, Jiangsu 215000, China

**Keywords:** TYRO3, colorectal cancer, drug-resistance, ENO1, EMT

## Abstract

Colorectal cancer (CRC) is a leading cause of cancer mortality worldwide. Although considerable advances in CRC treatment have been achieved, effective treatment improvement has hit a bottleneck. This study demonstrated that TYRO3 expression was aberrantly increased in CRC tissues with prognosis association. The prediction model of prognosis for CRC patients was constructed based on TYRO3 expression. The model suggested that the TYRO3 level is crucial to the final prediction results. We observed that knockdown TYRO3 expression could inhibit the proliferation and migration ability and reverse the drug resistance by constructing drug-resistant CRC cell lines. *In vivo* experiments also confirmed this conclusion. Thus, targeting TYRO3 combined with 5-Fu treatment could provide a better therapeutic effect. Additionally, TYRO3 could inhibit the EMT process by down-regulating ENO1, which may be achieved by interfering with energy metabolism in cancer cells. Therefore, the current study provides a theoretical basis for TYRO3 in drug-resistance of CRC cells and highlights a new strategy for CRC-targeted therapy.

## INTRODUCTION

Colorectal cancer (CRC) is one of the most common forms of gastrointestinal malignancies, the fourth most common type of cancer, and the fifth leading cause of cancer-related mortality [1, 2]. In recent years, the advancement of endoscopic resection, surgical resection, preoperative systemic treatment, local ablation treatment, palliative chemotherapy, immunotherapy, and targeted treatment has significantly improved the survival of CRC patients. However, drug resistance is a common phenomenon affecting the effectiveness and persistence of cancer treatment in CRC patients [3]. The drug resistance mechanism is usually the multiple effects of tumor burden, growth dynamics, physical barriers, immune system, and tumor microenvironment [4–6]. Thus, reversing drug resistance and enhancing the effect of chemotherapy and targeted therapy are essential in treating CRC.

The TAM family (TYRO3, AXL, MerTK) of receptor tyrosine kinases (RTK) is a crucial regulator of biological processes, such as cell growth, survival, and differentiation [7, 8]. RTK is established as a proto-oncogene in an early stage, and its aberrant expression correlates with tumor progression across many cancers. Recent studies have increased describing the role of three kinds of TAMs in tumorigenesis and tumor growth [9, 10]. Most studies have focused on the aberrant expression of AXL in various cancers. AXL promotes tumor growth and dissemination by positively affecting cell survival, proliferation, migration, and invasion. In addition, AXL is also associated with cell differentiation, vascular protection, apoptotic cell elimination, and regulating proinflammatory cytokine production [11–13]. Meanwhile, MerTK could mediate the phagocytosis of apoptotic cells [14, 15]. However, there are only a few studies on TYRO3, especially in tumor diagnosis and treatment [16]. Thus, TYRO3 expression level and its effect on tumorigenesis and drug resistance demand further clarification.

The TAM (TYRO3, AXL, MerTK) subfamily is prominent in multiple cancer types resistant to various therapeutic agents, such as targeted and conventional chemotherapy. Our study constructed CRC drug-resistant cell lines. We observed that TYRO3 expression was significantly increased, and the epithelial-mesenchymal transformation (EMT) process, wherein epithelial cells are transformed into mesenchymal phenotypic cells through a specific procedure, was elevated in drug-resistant CRC cells. Moreover, inhibiting TYRO3 expression can significantly inhibit the EMT process in tumor cells and reverse the drug resistance in CRC cells. Therefore, the current study explored the correlation between TYRO3 and drug resistance in CRC and the potential regulatory mechanism. This would provide a theoretical basis for reversing tumor cell drug resistance using TYRO3 targeted therapy, a new strategy for treating CRC.

## MATERIALS AND METHODS

### CRC specimens

In this study, we enrolled 120 CRC patients treated in the First Affiliated Hospital of Soochow University from 2015 to 2016. None of the patients underwent radiotherapy or chemotherapy before radical surgery. CRC and paired adjacent normal colon tissues were immediately collected after surgical resection and confirmed through histopathology. Personal or telephonic interviews were used to follow up with the patients for 60 months, and the time point was set as the date of CRC-related death or 60 months post-surgery. All the patients provided written informed consent. The independent ethics committees of the First Affiliated Hospital of Soochow University (2019100) and the Affiliated Suzhou Hospital of Nanjing Medical University (2021240) approved the study.

### Immunohistochemistry (IHC)

CRC and paired normal colorectal tissues were fixed using formalin, embedded in paraffin, cut into 5 μm sections, and stained using IHC [17]. The sections were incubated using the anti-TYRO3 (Abcam, UK) and anti-ENO1 (Boster, China) antibodies at 1:100 dilution for 2 hours at room temperature. TYRO3 expression in tissues was visualized with a tissue staining kit (Zhongshan Biotechnology, China). Two authors with expertise in pathology assessed the staining scores. IHC score was determined by the intensity multiple (0, negative; 1, weak; 2, moderate; 3, strong) and extent (0, 0–5%; 1, 6–25%; 2, 26–50%; 3, 51–75%; 4, >75%) score. We considered a final score of 0 as −; 1–4 as +; 5–8 as ++; 9–12 as +++. In this study, ++ or +++ was considered a positive expression, and – or + a negative [18].

### Cell culture

CRC cell lines, including HCT116, SW620, HT29, RKO, and LOVO, were obtained from the Central Laboratory of the Affiliated Suzhou Hospital of Nanjing Medical University. We constructed 5-Fu resistant HCT116 (H-DR) and HT29 (T-DR) cells by gradually increasing 5-Fu concentration to treat wild-type HCT116 (H-WT) and HT-29 (T-WT) within six months. The cells were grown in RPMI Medium 1640 (Hyclone, USA) with 10% FBS (Gibco, USA), 100 units/ml penicillin G sodium, and 100 μg/ml streptomycin sulfate (Gibco, USA). The cell lines were cultured under 5% CO_2_ at 37°C.

### Transfection of shRNA

The cells were grown to 80% confluency and transfected using a lentivirus TYRO3-shRNA technique (target sequences CAAAGAGATGTCCTTGTAATA) based on the manufacturer’s instructions [19]. The transfected cells were selected using G418 (Roche, Switzerland). Then, we selected clones with a stable TYRO3 knockdown for further experiments.

### Western blotting analysis

Protein samples were gathered in a lysis buffer with protease inhibitors. Sodium dodecyl sulfate-polyacrylamide gel electrophoresis (SDS-PAGE) was utilized for protein separation. Then, the samples were transferred to polyvinylidene fluoride (PVDF) membranes incubated with a specific antibody [17]. The Enhanced Chemiluminescence System (Invitrogen, CA) was used to detect the Protein strip, which was semi-quantified using ImageJ (NIH, USA). This study included the primary antibodies of anti-TYRO3 (1:1000, Abcam, UK), anti-ENO1 (1:1000, Boster, China), anti-E-cadherin (1:1000, Bioss, China), anti-Vimentin (1:1000, Bioss, China) and anti-β-Actin (1:5000, Bioss, China). ImageJ software helped analyze the protein quantity.

### Cell viability assay

The Cell Counting Kit-8 (CCK-8) assay (APExBIO, Houston, TX, USA) or the MTT assay kit (Amresco, Boise, ID, USA) could detect cell viability based on the manufacturer’s instructions using 96-well plates with 2000 cells per well.

### Colony formation assay

About 1000 cells were cultured in each well in the six-well plates for 10 days. The formed clones were fixed using methanol, stained using crystal violet solution, and counted under a microscope.

### Transwell invasion assay

The cells were seeded into the upper chambers with a density of 10,000 cells/200 μl in a serum-free medium. Moreover, the lower chambers were filled with 800 μl complete medium per well. After 12 hours of incubation, the filters were fixed with 4% paraformaldehyde, stained with 0.1% crystal violet, and the average of five random fields per sample was calculated.

### ATP measurement

Based on the manufacturer’s instructions, relative ATP levels were measured using an enhanced ATP Assay Kit (Beyotime, China). Total ATP levels were determined from the luminescence signals and normalized using the protein concentrations.

### Murine xenograft assay

SPF male BALB/c nude mice (four weeks, 16–18 g) were obtained from the Shanghai SLRC Laboratory Animal Co., Ltd. (Shanghai, China). The mice were randomly divided into each group (*n* = 5 per group) and injected subcutaneously with 5 × 10^6^ H-DR cells into the right dorsal flank on day 0 after acclimatizing for one week. The 5-Fu-treated mice were injected intraperitoneally with 50 mg/kg twice weekly. The Animal Ethics Committee of the Affiliated Suzhou Hospital of Nanjing Medical University (Suzhou, China) approved all the animal experiments.

### Statistical analysis

Data were represented as mean ± SD for at least three independent experiments. The statistical analyses were performed using SPSS 22.0 software (SPSS Inc., Chicago, IL, USA), GraphPad Prism 8 (San Diego, CA, USA), and R programs (version 3.6.1 for Windows, http://cran.r-project.org/). *T*-test (unpaired, two-tailed) or Mann–Whitney *U* test helped compare the means between groups. The IHC results were analyzed using Chi-square or Fisher’s exact tests. *P*-value < 0.05 was considered statistically significant.

### Data availability

Data will be made available on request.

## RESULTS

### TYRO3 is an aberrant increase in CRC tissues

**Figure 1:**
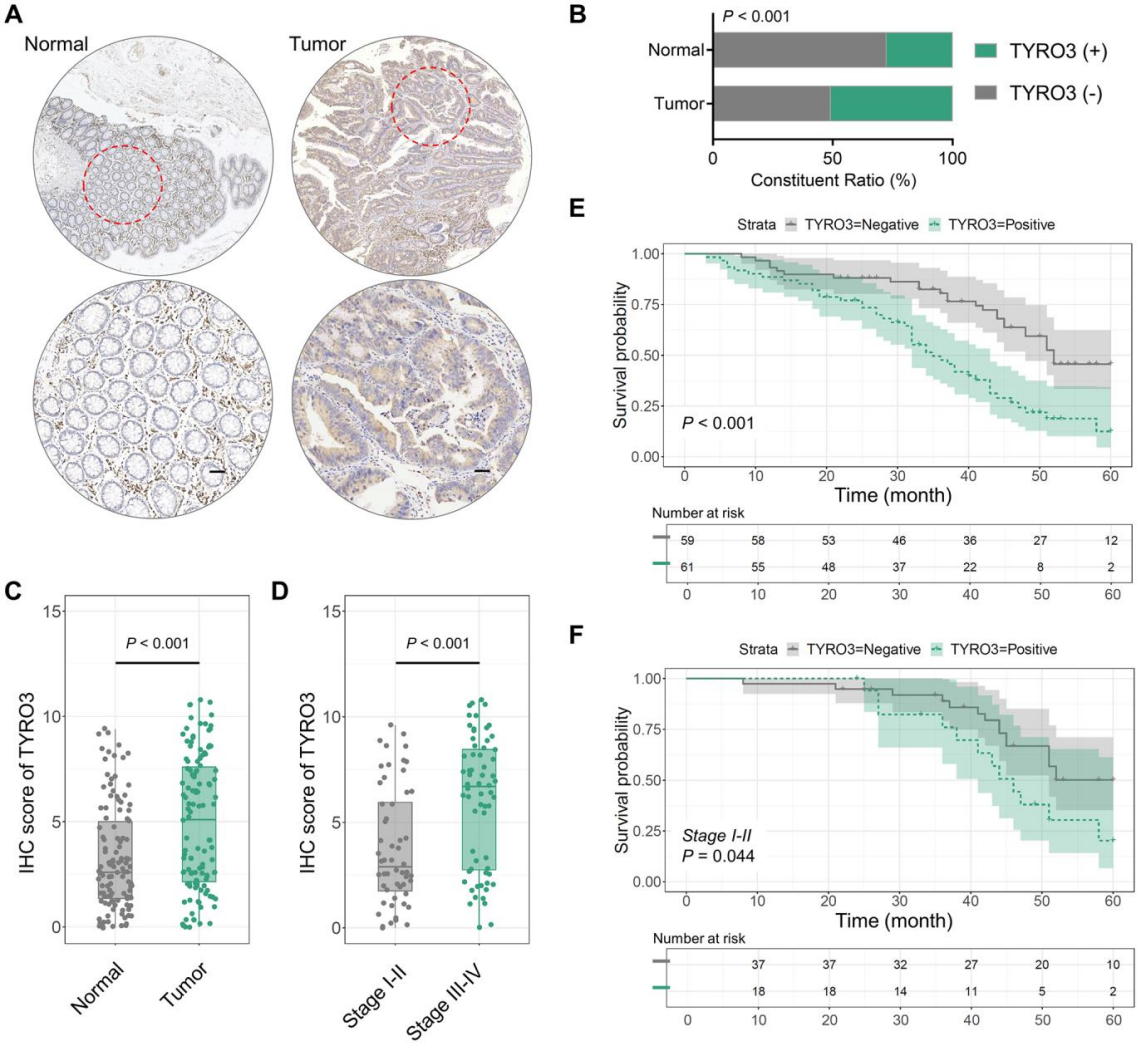
**TYRO3 expression in 120 CRC and paired normal tissues.** (**A**) Representative IHC images showing TYRO3 expression in CRC and paired normal tissues (scale bar = 100 μm). (**B**) TYRO3 positive ratio in tumor tissues and normal tissues. (**C**, **D**) IHC scores of TYRO3 in (**C**) tumor versus normal tissues, and (**D**) TNM stage I-II versus stage III-IV. (**E**) Overall survival analysis of TYRO3^pos^ versus TYRO3^neg^ CRC patients. (**F**) In subgroup of TNM staging I-II, overall survival analysis of TYRO3^pos^ versus TYRO3^neg^ CRC patients.

TYRO3 expression was detected in the cancer tissues of 120 CRC patients using the IHC test. It was observed that TYRO3 was significantly increased in CRC tissues compared to adjacent normal tissues (Figure 1A). Subsequently, IHC scores were calculated on 120 cancer and adjacent normal tissues and divided into TYRO3^pos^ and TYRO3^neg^ based on the results. A positive TYRO3 proportion in CRC tissues was significantly elevated than in normal tissues (*P* < 0.001, Figure 1B). We also analyzed the difference in IHC score between cancer and normal tissues, which showed a more significant TYRO3 expression in tumor tissues (*P* < 0.001, Figure 1C).

After clarifying the difference in TYRO3 expression between tumor and normal tissues, we further assessed whether TYRO3 expression in cancer tissues differed with the TNM stage alteration. IHC scores indicated that TYRO3 expression level was significantly higher in tumor tissues of CRC patients with TNM stage III-IV than those with TNM stage I-II (*P* < 0.001, Figure 1D). Thus, aberrant TYRO3 expression levels increased in CRC tissues gradually with disease progression. This provides a theoretical basis for diagnosing the occurrence and development of CRC depending on TYRO3 expression in tissues.

### TYRO3 expression in CRC tissues is closely associated with the clinicopathological parameters and predicts poor survival

The correlation between TYRO3 expression and clinicopathological indicators based on negative or positive IHC staining scores of TYRO3 in CRC tissues was also evaluated. High TYRO3 expression was associated with neural invasion (*P* = 0.019), lymph node metastasis (*P* < 0.001), and TNM stage (*P* < 0.001). However, there was no correlation with age, gender, tumor size, differentiation degree, venous invasion, and tumor invasion depth (*P* > 0.05, Table 1).

**Table 1:** Relationship between TYRO3 and clinic-pathological factors in CRC patients.

**Variables**	**TYRO3**		
	**Negative**	**Positive**	***P* value**
Age (years)			
≤60	29	22	0.147
>60	30	39	
Gender			
Male	29	30	0.998
Female	30	31	
Tumor size (cm)			
<5	28	27	0.725
≥5	31	34	
Degree of differentiation			
Well	37	46	0.132
Poor	22	15	
Venous invasion			
Negative	35	37	0.881
Positive	24	24	
Neural invasion			
Negative	44	33	0.019^*^
Positive	15	28	
Depth of tumor invasion			
T1-2	23	19	0.368
T3-4	36	42	
Lymph node metastasis			
No	38	18	<0.001^*^
Yes	21	43	
TNM staging			
I-II	38	18	<0.001^*^
III-IV	21	43	

^*^P < 0.05.

Lymph node metastasis and TNM stage are clinicopathological indicators associated with the survival of CRC tumorigenesis patients [18]. Therefore, the predictive effect of TYRO3 high expression was analyzed on the survival of patients. Firstly, TYRO3 positive indicated poor prognosis using the Kaplan-Meier curve (*P* < 0.001, Figure 1E). In the TNM stage I-II subgroup, TYRO3^pos^ patients also showed poor survival (*P* = 0.044, Figure 1F). Thus, TYRO3 expression can predict patient survival in early-stage CRC patients.

**Figure 2:**
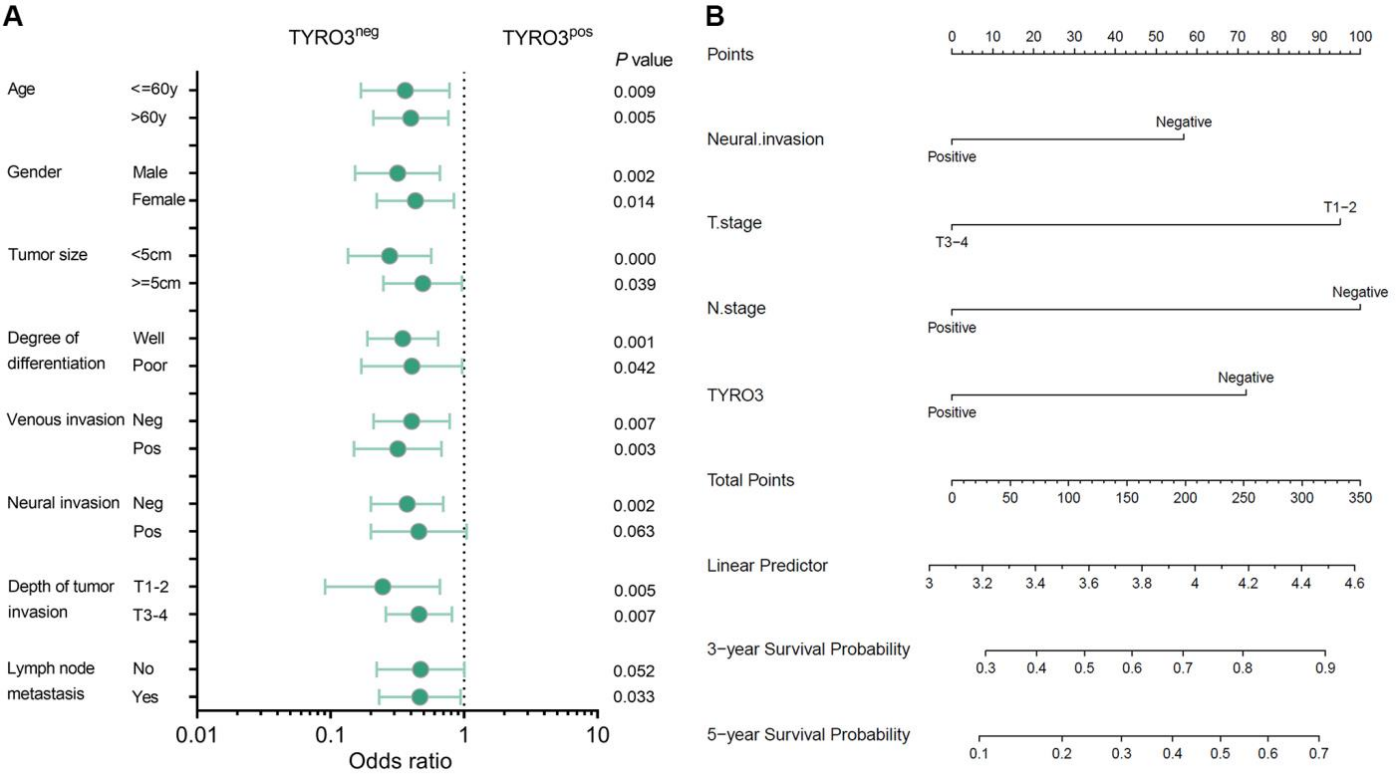
**Subgroup analysis of prognosis and construction of prognostic prediction model.** (**A**) Overall survival analysis of TYRO3^pos^ vs. TYRO3^neg^ CRC patients in subgroups demarcated according to each clinicopathological indicators. (**B**) Nomograms to predict overall survival of CRC patients. Points of each variable were obtained via a vertical line between each variable and the point scale. The predicted survival rate was correlated with the total points by drawing a vertical line from the total points scale to the overall survival.

Subsequently, whether TYRO3 expression could predict the prognosis of CRC patients in each clinicopathological index subgroup was also analyzed. The subgroup analysis revealed that TYRO3^pos^ predicted poor prognosis irrespective of age, gender, tumor size, differentiation degree, venous invasion, and tumor invasion depth (*P* < 0.05, Figure 2A). Moreover, TYRO3^pos^ correlated with poor survival in patients having negative neural invasion or positive lymph node metastasis (*P* < 0.05, Figure 2A). However, no statistical correlation could be observed between the TYRO3 expression level and survival prognosis in patients having positive neural invasion or negative lymph node metastasis (*P* > 0.05, Figure 2A). This evidence provides a scope for the TYRO3 expression level to determine the prognosis of CRC patients.

### A prognostic prediction model based on TYRO3 expression

The correlation between the TYRO3 expression level in tumor tissues and the overall survival time of CRC patients was considered to determine the potential role of TYRO3 expression in prognosis prediction. Therefore, a prognostic prediction model was constructed for CRC patients depending on TYRO3 expression. At first, the univariate analyses of postoperative survival in patients were performed using Cox’s proportional hazard model. The results revealed that neural invasion, depth of tumor invasion, lymph node metastasis, and TYRO3 expression were independent risk factors (*P* < 0.05, Table 2). Then, multivariate analysis using these four independent risk factors demonstrated that depth of tumor invasion, lymph node metastasis, and TYRO3 expression significantly affected survival (*P* < 0.05, Table 2).

**Table 2:** Results of univariate and multivariate analyses of postoperative patients' survival by Cox's proportional hazard model.

**Varieties**	***n***	**Univariate analysis**			**Multivariate analysis**		
		**HR**	**95% CI**	***p* value**	**HR**	**95% CI**	***p* value**
Age (≤60 or >60 years)	51/69	0.712	0.441–1.151	0.166			
Gender (Male/Female)	59/61	0.857	0.537–1.369	0.519			
Tumor size (<5 or ≥5 cm)	55/65	0.991	0.621–1.582	0.971			
Degree of differentiation (moderate-well/poor)	83/37	1.014	0.613–1.679	0.955			
Venous invasion (negative/positive)	72/48	0.719	0.450–1.148	0.167			
Neural invasion (negative/positive)	77/43	0.543	0.337–0.875	0.012^*^	0.639	0.393–1.040	0.072
Depth of tumor invasion (T1-2/T3-4)	42/78	0.469	0.276–0.795	0.005^*^	0.538	0.316–0.915	0.022^*^
Lymph node metastasis (negative/positive)	56/64	0.407	0.250–0.661	<0.001^*^	0.519	0.311–0.866	0.012^*^
TYRO3 expression (negative/positive)	59/61	0.372	0.227–0.608	<0.001^*^	0.493	0.294–0.829	0.008^*^

^*^P < 0.05.

Based on the results from the multivariate analysis in Cox’s proportional hazard model, the three factors of tumor invasion, lymph node metastasis, and TYRO3 expression were included. They were combined with the follow-up information of CRC patients to construct a prediction model represented by nomograms (Figure 2B). The nomograms could predict the 3- and 5-year overall survival of CRC patients. The predicted survival rate was associated with the total points by forming a vertical line from the total points scale to the overall survival. Therefore, the status of tumor invasion, lymph node metastasis, and TYRO3 expression significantly affected the total points, suggesting they had essential roles in predicting CRC patient prognosis.

### The association between drug resistance and TYRO3 expression level in CRC

**Figure 3:**
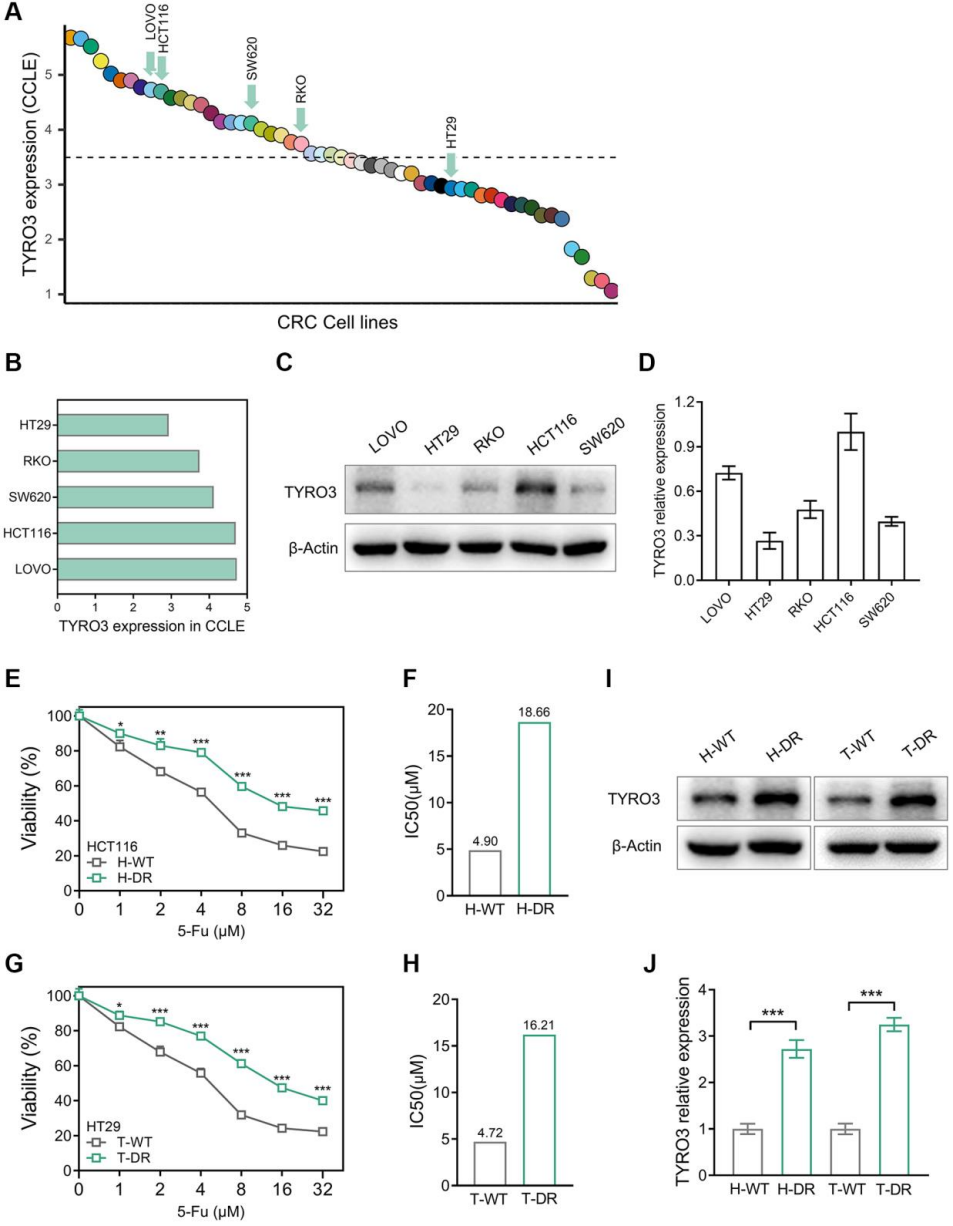
**Construction of drug-resistant cell lines and alteration of TYRO3 expression.** (**A**) TYRO3 expression in CRC cell lines searched from the CCLE platform. (**B**) TYRO3 expression in 5 CRC cell lines selected by CCLE platform. (**C**) TYRO3 expression in 5 CRC cell lines evaluated by Western blot. (**D**) Immunoblot result of TYRO3 expression in 5 CRC cell lines quantified by ImageJ. (**E**) The viability of H-WT and H-DR treated with different concentrations of 5-Fu for 24 hours. (**F**) The IC50 of H-WT and H-DR treated with 5-Fu. (**G**) The viability of T-WT and T-DR treated with different concentrations of 5-Fu for 24 hours. (**H**) The IC50 of T-WT and T-DR treated with 5-Fu. (**I**) Western blot detecting the change of TYRO3 protein levels in wild type and drug-resistance CRC cells. (**J**) Immunoblot result of wild type and drug-resistance CRC cells semi-quantified by ImageJ. ^*^P < 0.05, ^**^P < 0.01, ^***^P < 0.001.

Drug resistance in tumor cells is the challenging point of tumor therapy. Previous studies have observed that the TYRO3 expression level is closely related to drug resistance [20, 21]. We attempted to construct CRC drug-resistant cell lines using 5-Fu to clarify this potential correlation and mechanism. The expression level of TYRO3 in CRC cell lines was evaluated by the CCLE platform, and related cell lines were compared (Figure 3A, 3B). CCLE platform indicated that the expression level of TYRO3 in HCT116 and LOVO cell lines was relatively high, while that in HT29 cell lines was relatively low. Subsequently, the expression level of TYRO3 in these five cell lines was investigated. Later, HCT116, with a relatively high expression of TYRO3, and HT29, with a relatively low expression, were selected for the next experiment (Figure 3C, 3D).

The drug resistance of H-DR and T-DR was also assessed. For HCT116 cell lines, the IC50 level of H-DR was higher than H-WT after cells were treated with different concentrations of 5-Fu (Figure 3E, 3F). Similarly, the IC50 level of T-DR was significantly higher than T-WT in the HT29 cell line (Figure 3G, 3H). Then, the expression level of TYRO3 was detected in H-DR and T-DR cell lines. Interestingly, the TYRO3 expression level in H-DR and T-DR cells was significantly elevated compared to wild-type cell lines (Figure 3I, 3J). Thus, the expression level of TYRO3 gradually increased with the improvement of drug resistance in CRC cells, indicating a potential correlation.

### Inhibition of TYRO3 expression reversing drug resistance in CRC cells

**Figure 4:**
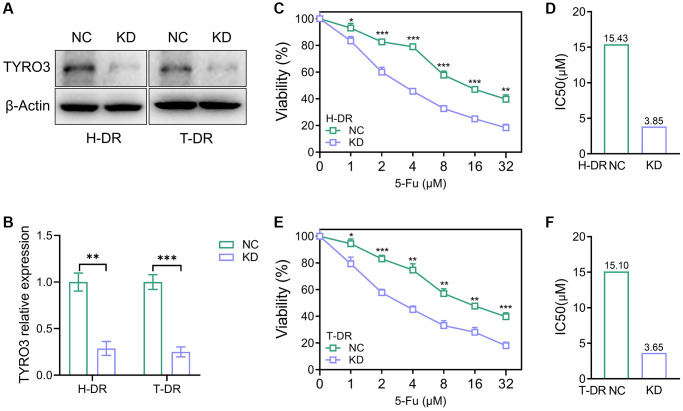
**Reverse of drug-resistant cells caused by knockdown of TYRO3 expression.** (**A**) Western blot assessing the effect of TYRO3-shRNA on H-DR and T-DR cells (**B**) Immunoblot result of H-DR and T-DR semi-quantified by ImageJ. (**C**) The viability of NC and TYRO3-KD H-DR cells treated with different concentrations of 5-Fu for 24 hours. (**D**) The IC50 of NC and TYRO3-KD cells treated with 5-Fu. (**E**) The viability of NC and TYRO3-KD T-DR cells treated with different concentrations of 5-Fu for 24 hours. (**F**) The IC50 of NC and TYRO3-KD cells treated with 5-Fu. Abbreviations: NC: negative control; KD: TYRO3-shRNA. ^*^P < 0.05, ^**^P < 0.01, ^***^P < 0.001.

TYRO3-shRNA was used to knockdown the expression level of TYRO3 to explore its expression alteration effect on drug resistance of CRC cells (Figure 4A). The results indicated that TYRO3 expression in H-DR and T-DR cells was significantly inhibited (Figure 4B). After inhibiting TYRO3 expression in H-DR cells, the drug-resistance was reversed and IC50 was significantly reduced than in NC group cells (Figure 4C, 4D). TYRO3 expression inhibition in T-DR cells could enhance drug sensitivity (Figure 4E, 4F).

**Figure 5:**
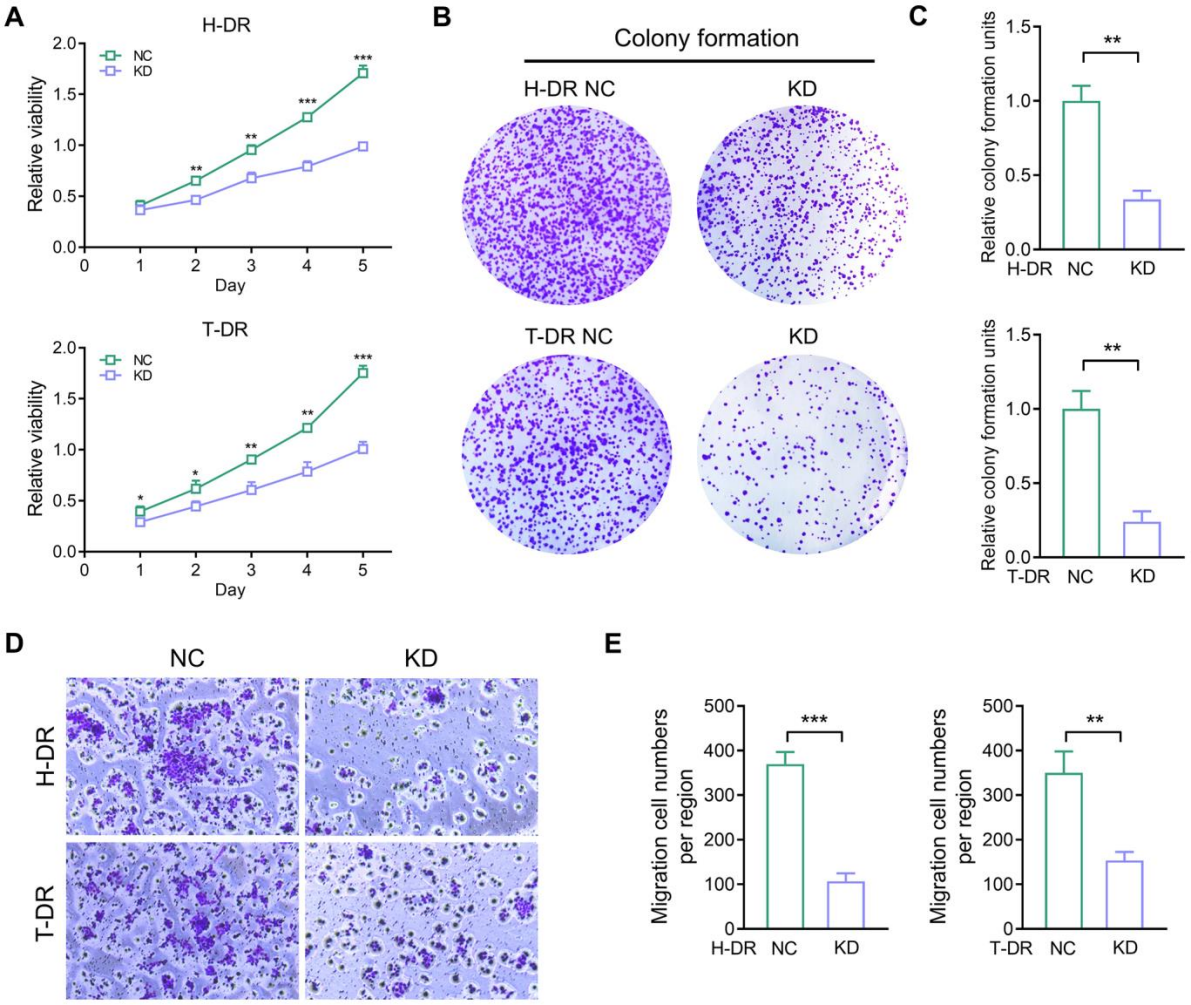
**The proliferation and migration ability in drug-resistant cells were inhibited by down-regulating TYRO3 expression.** (**A**) Proliferation ability of H-DR and T-DR transfected with TYRO3-shRNA assessed by CCK-8 assay. (**B**) Colony formation capacity of H-DR and T-DR transfected with TYRO3-shRNA assessed. (**C**) Colony formation units counted. (**D**) Migration capacity of H-DR and T-DR transfected with TYRO3-shRNA assessed. (**E**) Migration cell numbers counted. Abbreviations: NC: negative control; KD: TYRO3-shRNA. ^*^P < 0.05, ^**^P < 0.01, ^***^P < 0.001.

Several studies depicted that the aberrant TYRO3 expression in tumor cells effectively enhances the proliferation and migration of tumor cells [22, 23]. Therefore, the expression of TYRO3 would be aberrantly activated with tumorigenesis and metastasis. Then, the aberrant TYRO3 would promote the proliferation and metastasis ability of cells. This study constructed CRC drug-resistant cell lines for TYRO3 expression knockdown. In drug-resistant cell lines H-DR and T-DR, knockdown of TYRO3 also suppressed the proliferation ability in tumor cells (Figure 5A). Meanwhile, inhibiting TYRO3 expression also limited the clone formation and migration ability of drug- resistant cells (Figure 5B–5E). Therefore, targeting TYRO3 is a potentially effective treatment strategy even in drug-resistant tumor cells.

### TYRO3 promotes the effect of ENO1 and the process of EMT

The proliferation and migration of tumor cells are closely associated with energy generation and utilization [24]. Inhibiting TYRO3 expression could effectively reverse drug resistance and down-regulate the proliferation and migration ability of cancer cells. Therefore, the correlation between TYRO3 expression and tumor metabolic pathway was further explored to determine its effect on metabolism.

**Figure 6:**
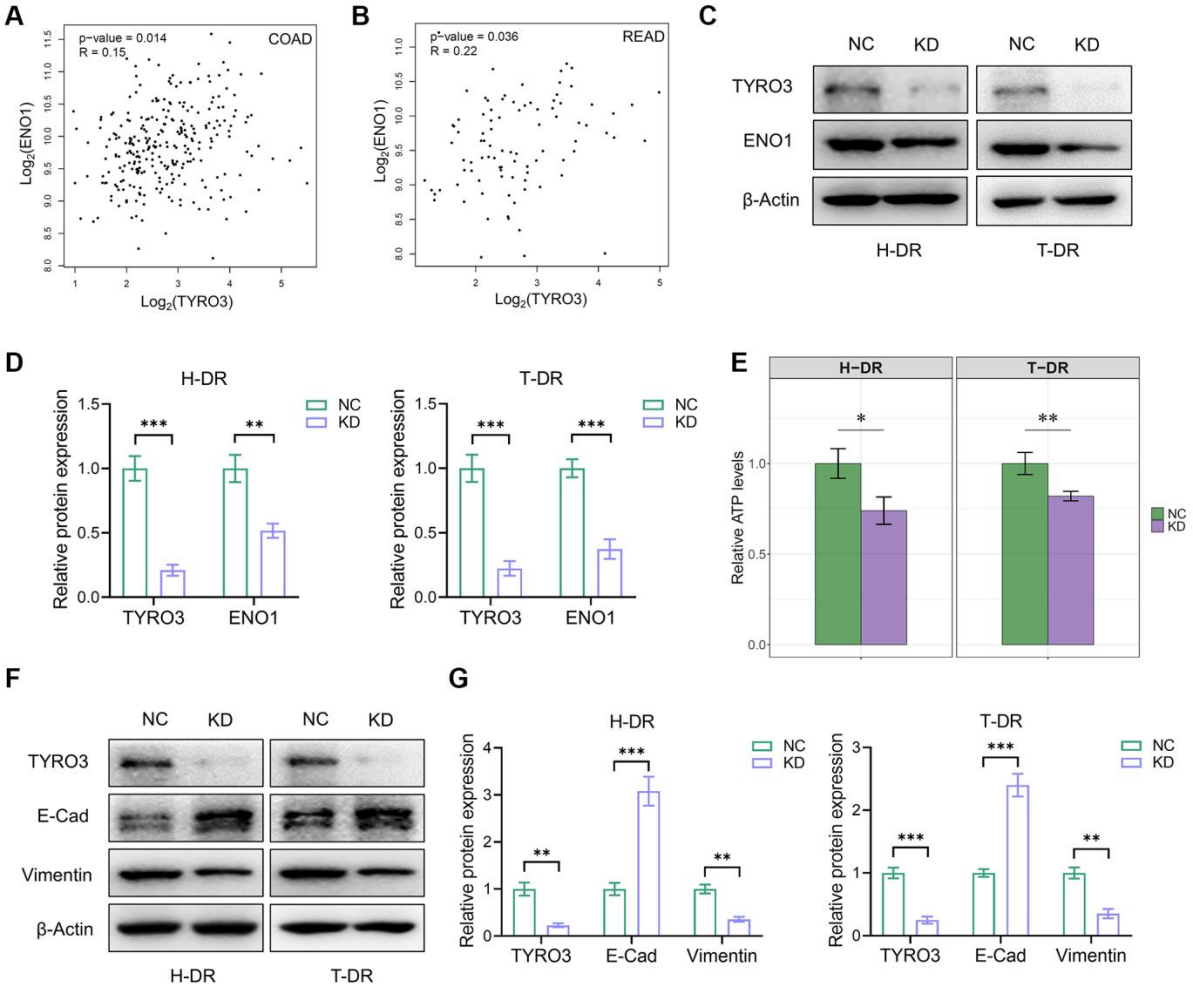
**TYRO3 regulated the effect of ENO1 and the process of EMT in CRC cells.** (**A**, **B**) Correlation analysis of TYRO3 and ENO1 gene expression levels in (**A**) colon cancer and (**B**) rectal cancer patients in TCGA datasets via GEPIA platform. (**C**) Western blot showing TYRO3, ENO1 and β-Actin protein levels in H-DR and T-DR cells transfected with TYRO3-shRNA or negative control. (**D**) Immunoblot result of H-DR and T-DR semi-quantified by ImageJ. (**E**) Comparisons of relative ATP levels in H-DR and T-DR cells transfected with TYRO3-shRNA or negative control. (**F**) Western blot showing TYRO3, E-Cadherin, Vimentin and β-Actin protein levels in H-DR and T-DR cells transfected with TYRO3-shRNA or negative control. (**G**) Immunoblot result of H-DR and T-DR semi-quantified by ImageJ. Abbreviations: NC: negative control; KD: TYRO3-shRNA. ^*^P < 0.05, ^**^P < 0.01, ^***^P < 0.001.

Firstly, the association between the expression of TYRO3 and GLUT1, GLUT2, and GLUT3 in CRC tissues in TCGA database was analyzed using the GEPIA platform (Supplementary Figure 1A–1F). Although TYRO3 expression was positively correlated with GLUT1 and GLUT3 expression in rectal cancer tissues, no such correlation could be observed with GLUT1, GLUT2, and GLUT3 expression in colon cancer tissues. Our previous findings on the role of ENO1 in tumorigenesis and tumor metabolism enabled the correlation analysis between TYRO3 and ENO1 expression [25]. The results indicated that TYRO3 expression was positively associated with ENO1 expression in colon and rectal cancer tissues from TCGA database (Figure 6A, 6B).

Subsequently, the expression level of TYRO3 in H-DR and T-DR was reduced. Moreover, the ENO1 expression level was also significantly inhibited (Figure 6C, 6D), causing a significant decrease in cellular ATP levels (Figure 6E). Therefore, TYRO3 expression inhibition in drug-resistant cells may reduce ENO1 expression, down-regulating the proliferation and migration of tumor cells by restricting glucose utilization and partial ATP production. EMT process-related targets were detected in H-DR and T-DR cells to demonstrate the effect of TYRO3 on tumor cells (Figure 6F, 6G). Thus, inhibiting the expression of TYRO3 in H-DR and T-DR could promote the expression of E- Cadherin and reduce the expression of Vimentin, suggesting the inhibition of the EMT process of tumor cells.

### Suppressing TYRO3 expression enhances the anticancer effect of 5-Fu *in vivo*

**Figure 7:**
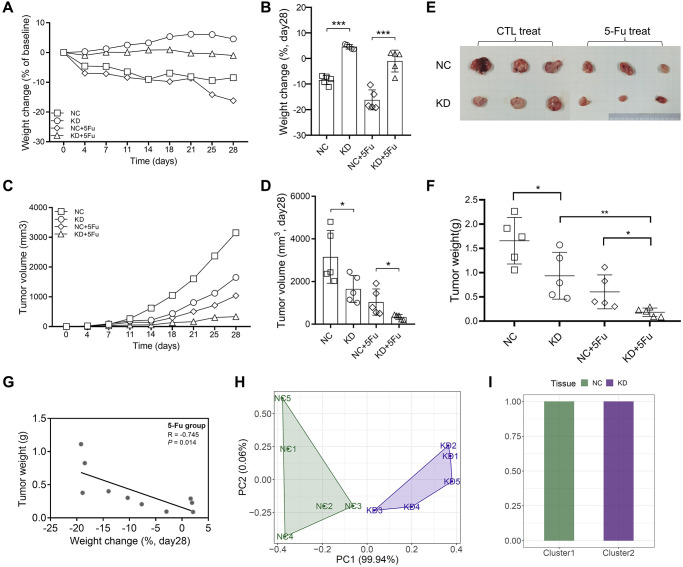
**Inhibition of TYRO3 expression enhances drug sensitivity of CRC cells *in vivo*.** (**A**) Weight change of the NC, KD, NC+5-Fu and KD+5-Fu group mice recorded twice a week during the experiment (*n* = 5). (**B**) Weight change of the NC, KD, NC+5-Fu and KD+5-Fu group mice on day28 (*n* = 5). (**C**) Tumor volume of the NC, KD, NC+5-Fu and KD+5-Fu group mice recorded twice a week during the experiment (*n* = 5). (**D**) Tumor volume of the NC, KD, NC+5-Fu and KD+5-Fu group mice on day28 (*n* = 5). (**E**) Representative pictures of subcutaneous tumors harvested from NC and TYRO3-KD group treated with 5-Fu or not (the maximum and minimum removed). (**F**) Tumor weight of the NC, KD, NC+5-Fu and KD+5-Fu group mice (*n* = 5). (**G**) The association analysis between weight change of mice and tumor weight in 5-Fu treated group (*n* = 5). (**H**) Stratification of mice into different clusters according to weight change and tumor weight in 5-Fu treated group (*n* = 5). (**I**) Percentage of NC and TYRO3-KD mice in each cluster. Abbreviations: NC: negative control; KD: TYRO3-shRNA. ^*^P < 0.05, ^**^P < 0.01, ^***^P < 0.001.

We also used H-DR cells to construct a subcutaneous tumor model of mice. The mice were divided into four groups (*n* = 5): NC, KD, NC+5Fu, and KD+5Fu. The results showed that the nutritional status of mice in the TYRO3-KD group was significantly improved compared to the NC group (Figure 7A, 7B). 5-Fu treatment could decrease the overall weight of mice to a certain extent. However, this treatment did not affect the tendency that the body weight of the KD+5Fu group was higher than the NC+5Fu group.

The growth of subcutaneous tumors was recorded twice a week. The growth trend of subcutaneous tumors revealed that the knockdown of TYRO3 expression inhibits tumor growth, and TYRO3-KD combined with 5-Fu could provide a better inhibitory effect (Figure 7C, 7D). The subcutaneous tumor was harvested after four weeks of subcutaneous tumor construction. The results also indicated that TYRO3-KD combined with 5-Fu could inhibit tumor growth significantly (Figure 7E, 7F).

Then, the NC+5Fu and KD+5Fu groups were analyzed to clarify whether inhibiting TYRO3 expression in 5-Fu-treated mice enhanced the therapeutic effect *in vivo*. For the two mice groups included in the analysis, the nutritional status of those with bigger tumors decreased significantly (Figure 7G). A significant difference in tumor and body weights due to TYRO3 expression was observed between the NC and KD groups in the PC1 axis. Thus, cluster analysis of mice could effectively distinguish the NC group from the TYRO3-KD group (Figure 7H, 7I). Therefore, the tumor growth in the TYRO3-KD group was significantly slower than in the control group when the mice were treated with 5-Fu. Moreover, the nutritional status of mice was better, indicating that targeting TYRO3 could enhance the antitumor effect of 5-Fu.

## DISCUSSION

Considerable advances in the mechanism and treatment of CRC have been achieved in recent years [1, 2, 26]. Despite the effective improvement and the significant extension of patient survival, the treatment has hit a bottleneck. The heterogeneity of cancer etiology is an important reason for poor treatment results [17, 27]. Thus, expanding our understanding of CRC pathogenesis and development can improve its treatment.

The current study observed that the expression of TYRO3, a member of the unique RTK family, was significantly elevated in CRC than in normal tissues. The aberrant TYRO3 expression is closely associated with neural invasion, lymph node metastasis, and TNM stage, thereby predicting poor prognosis. The survival follow-up information of CRC patients and TYRO3 expression level in cancer tissues were utilized to conduct relevant subgroup analysis depending on clinicopathological indicators. The results indicated that TYRO3^pos^ was correlated with poor survival in patients with negative neural invasion or positive lymph node metastasis. However, no statistical correlation between TYRO3 expression level and survival prognosis metastasis could be observed in patients with positive neural invasion or negative lymph node. Thus, the applied scope of TYRO3 expression could assess the prognosis of CRC patients.

The TYRO3, AXL, and MER members from the TAM family of RTKs were the latest to be identified [8]. TAM RTKs are ectopically induced or overexpressed in various human cancers, promoting cancer cell survival, chemotherapy resistance, migration, and invasion [28]. Despite being associated with many malignancies, most research has focused on the typical RTK family, such as EGFR and VEGFR [29, 30]. This study also demonstrated that TYRO3 is associated with CRC occurrence and development, the prediction effect, and targeted tumor diagnosis and treatment. We attempted to construct a clinical prognosis prediction model based on TYRO3 expression to further explore its clinical application value. Neural invasion, depth of tumor invasion, lymph node metastasis, and TYRO3 expression were included using Cox univariate analysis. These four factors had an important impact on patient survival in multivariate analysis. Therefore, a survival prediction model was constructed based on these four factors, indicating a significant effect of TYRO3 has expression on the model. The results provided a theoretical basis for the clinical application of TYRO3 detection.

Chihiro Uejima et al. considered TYRO3 could mediate tumor progression and predict the prognosis of gastric cancer patients [31]. Dehu Chen found that TYRO3 expression was significantly elevated in gastric cancer tissues. TYRO3 facilitates cell growth and metastasis by activating the Wnt/β-catenin signaling pathway [32]. In colon cancer cells, C-W Chien also observed that high expression of TYRO3 could enhance tumor proliferation and migration [19]. Although TAMs are not potent oncogenes in solid and hematologic cancers, their overexpression results in resistance to conventional and targeted chemotherapy [27, 33, 34].

5-Fu helped construct drug-resistant cell lines H-DR and T-DR, and their drug-resistance ability was evaluated to determine the involvement of TYRO3 in the drug-resistance process of CRC. The results indicated that the IC50 level of H-DR was significantly higher than in H-WT and a similar result in T-DR, indicating the effectiveness of the two drug-resistant cell lines. Interestingly, the expression level of TYRO3 in both H-DR and T-DR was significantly elevated compared with wild-type cell lines. Subsequently, the expression level of TYRO3 was reduced in the cells, and the drug-resistance cells were reversed with TYRO3-KD compared with the NC group. Moreover, IC50 was significantly decreased, suggesting that inhibiting TYRO3 expression could improve its drug sensitivity.

Recent studies showed that TYRO3 was associated with EMT signatures [19, 27]. Gain-of-function and loss-of-function experiments were performed using H-DR and T-DR. The results demonstrated that the knockdown of TYRO3 could also inhibit the proliferation, clone formation, and migration ability of drug-resistant cells. Therefore, targeting TYRO3 is a potentially effective treatment even for drug-resistant tumor cells. Meanwhile, targeted inhibition of TYRO3 can suppress the EMT process by regulating EMT-related targets, including Vimentin and E-Cadherin.

The proliferation, migration, and EMT processes of CRC depend on energy generation and utilization. The inhibition of TYRO3 expression could effectively reverse its drug resistance and down-regulate cancer cell proliferation and migration. Therefore, the correlation between TYRO3 expression and tumor metabolic genes was also explored. TYRO3 expression in CRC tissue was positively correlated with the metabolically associated gene ENO1. Subsequently, we reduced the expression of TYRO3 in drug-resistant cell lines through GEPIA platform selection and verification. We observed that ENO1 expression level was also inhibited, significantly decreasing cellular ATP levels.

Glycolysis is an energy supply and metabolism mechanism among organisms [35–37]. The activation regulation of ENO1 affects glycolysis and embryonic stem cell differentiation [38]. Glycolytic control is crucial for the abnormal proliferation of malignant tumor cells [37]. The classic form of metabolic reprogramming in cancer cells is the alteration in glucose metabolism. Elevated expression of glucose transporters, such as Glut1/Glut3 on cell membranes, causes increased glucose consumption in cancer cells [39, 40]. Metabolic intermediates synthesized by glucose metabolism enhance the biosynthesis of nucleotides, amino acids, and triglycerides. Many key metabolic enzymes are out of control during this process, including ENO1 [41, 42]. TYRO3 may regulate the expression and activation of ENO1, affecting energy metabolism and utilization. Therefore, targeting TYRO3 could interfere with energy generation, hinder proliferation and migration ability, and improve drug resistance of tumor cells.

In summary, TYRO3 expression was aberrantly increased in CRC tissues, correlating with prognosis. The prediction model of the prognosis of CRC patients indicates that TYRO3 expression level significantly affects the final prediction results. We demonstrated that knockdown TYRO3 expression could inhibit the proliferation and migration ability of drug-resistant cells and reverse the drug resistance by constructing drug-resistant CRC cell lines. *In vivo* experiments confirmed this finding and indicated that TYRO3 targeting combined with 5-Fu could achieve a better therapeutic effect. Additionally, targeting TYRO3 can down-regulate ENO1 to inhibit the EMT process by interfering with energy metabolism in cancer cells.

## Supplementary Materials

Supplementary Figure 1
