# Supplementary material for: TYRO3 promotes tumorigenesis and drug resistance in colorectal cancer by enhancing the epithelial-mesenchymal transition process
Source: Aging (Albany NY). 2023 Apr 14;15(8):3035–51. doi: 10.18632/aging.204656 (PMC10188355; doi:10.18632/aging.204656)
Supplement: Supplementary Figure 1 [file aging-15-204656-s001.pdf]

SUPPLEMENTARY FIGURE

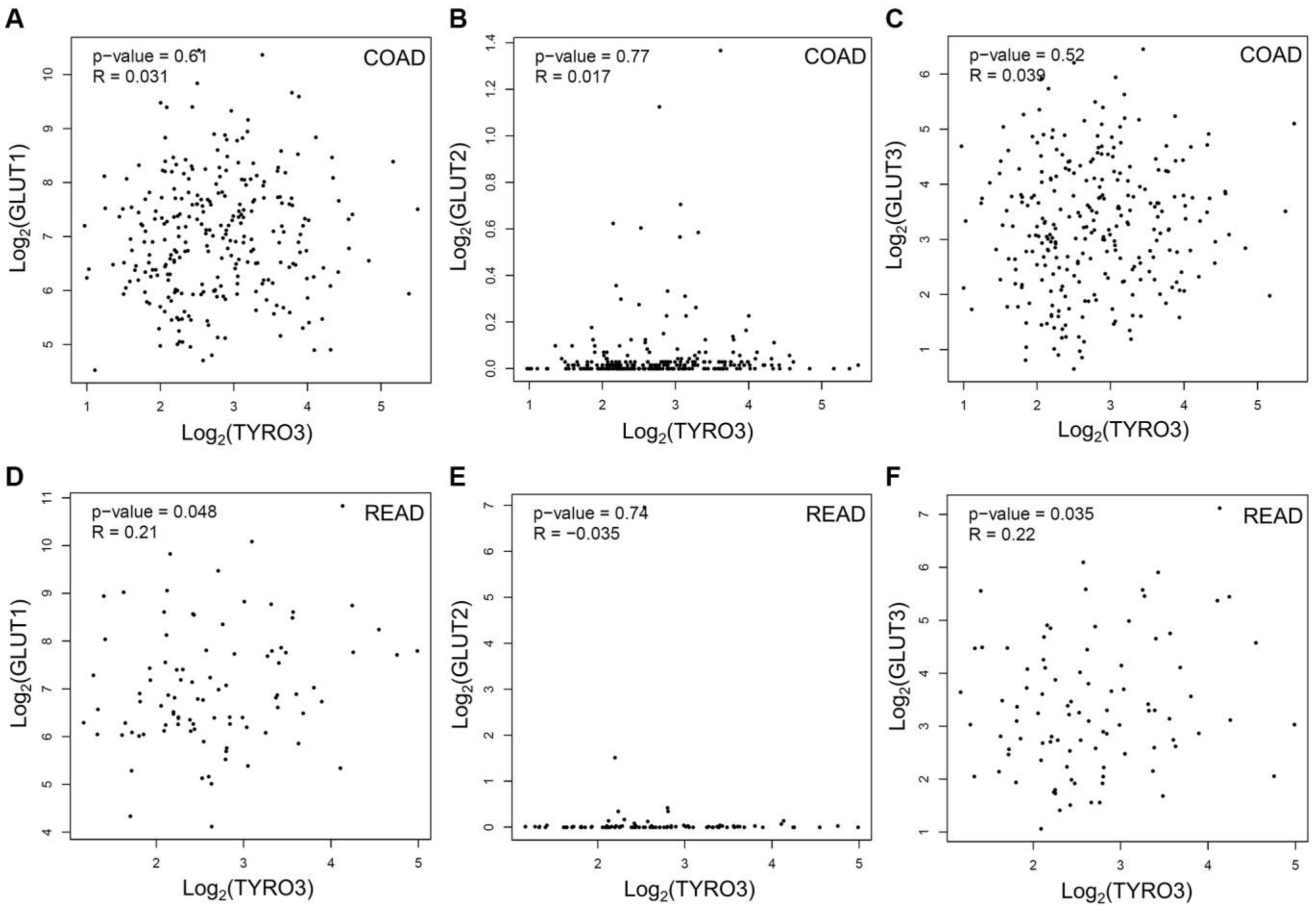

Supplementary Figure 1. Correlation between TYRO3 and GLUT expression in colorectal cancer tissues in TCGA database. (**A**–**C**) Correlation analysis of TYRO3 and (**A**) GLUT1, (**B**) GLUT2, (**C**) GLUT3 gene expression levels in colon cancer patients in TCGA datasets via GEPIA platform. (**D**–**F**) Correlation analysis of TYRO3 and (**D**) GLUT1, (**E**) GLUT2, (**F**) GLUT3 gene expression levels in rectal cancer patients in TCGA datasets via GEPIA platform. Abbreviations: COAD: colon cancer; READ: rectal cancer.
